# To: Perceptions of intensive care unit health care professionals in Brazil regarding postintensive care syndrome: a survey study

**DOI:** 10.62675/2965-2774.20260432

**Published:** 2026-05-05

**Authors:** Nav La, Schawanya K. Rattanapitoon, Chutharat Thanchonnang, Nathkapach K. Rattanapitoon

**Affiliations:** 1 International University Faculty of Medicine and Pediatrics Phnom Penh Cambodia Faculty of Medicine and Pediatrics, International University - Phnom Penh, Cambodia.; 2 FMC Medical Center Nakhon Ratchasima Thailand FMC Medical Center - Nakhon Ratchasima, Thailand.; 3 Mahidol University Nakhon Sawan Campus Faculty of Public Health Nakhon Sawan Thailand Faculty of Public Health, Mahidol University, Nakhon Sawan Campus - Nakhon Sawan, Thailand.

## Dear Editor,

The national survey by Teles et al.^(1)^ provides an important snapshot of how intensive care unit (ICU) professionals in Brazil perceive postintensive care syndrome (PICS) and PICS-family (PICS-F). However, when interpreted in context, the findings reveal a deeper structural issue that warrants explicit attention. Although professional awareness is relatively high, institutional capacity to act on that awareness remains markedly limited. This mismatch suggests that the central barrier to improving survivorship is not knowledge, but the absence of organizational mechanisms that anchor long-term outcomes within ICU and hospital governance.

The contrast is striking. While most respondents reported familiarity with PICS, only a minority indicated that their institutions conducted structured assessments, consistently used validated tools, or screened high-risk patients and families prior to ICU discharge.^(1)^ This gap mirrors observations from international survivorship research, in which long-term outcomes remain peripheral to institutional quality metrics, resulting in chronic under-implementation despite adequate clinician awareness.^(2)^ Thus, the findings of Teles et al.^(1)^ should be understood not simply as evidence of incomplete adoption of best practices, but as a systemic perception-action divide.

Methodological features of the survey reinforce this interpretation. Convenience sampling – largely from a national congress – likely oversampled highly engaged professionals, thereby inflating awareness. Physicians were also overrepresented, potentially obscuring perspectives of disciplines more directly involved in PICS prevention and rehabilitation. Furthermore, dichotomizing Likert scales may have restricted the detection of intermediate institutional priorities, particularly in resource-limited environments. These elements suggest that the institutional deficits identified in the study may, in fact, be underestimates.

To conceptualize this structural barrier, we propose a Perception-Action Gap model, wherein clinicians recognize the long-term consequences of critical illness; institutions lack operational pathways to address them; the lack of visibility into survivorship outcomes diminishes perceived urgency; and institutional deprioritization is perpetuated. Evidence linking ICU discharge processes, functional decline, and late mortality supports the relevance of this framework.^(3,4)^ Addressing this loop may require integrating post-ICU trajectories into hospital performance measures - a shift that realigns incentives with survivorship.

The undervaluation of PICS-F at the institutional level, also reported by Teles et al.,^(1)^ represents a missed opportunity. Family psychological distress influences patient recovery, adherence, and long-term quality of life.^(2,5)^ Embedding family assessment within discharge processes may therefore provide a pragmatic entry point for institutions seeking measurable improvements with modest resource investment.

The contribution by Teles et al.^(1)^ is timely, but its implications extend beyond mere awareness. Their data highlight a systemic gap between professional perception and institutional action - an issue central to strengthening post-ICU care in middle-income health systems. Future work should focus on structural reforms that align organizational priorities with the realities of survivorship.

## AI declaration

The authors used ChatGPT solely for language editing. All scientific content, interpretations, and conclusions are entirely the authors’ own work.
